# Iatrogenic Pituitary Shutdown: A Rare Adverse Event of Programmed Cell Death-Ligand 1 Inhibitor

**DOI:** 10.1210/jcemcr/luad157

**Published:** 2024-01-02

**Authors:** Mohammed Al-Hiari, Anthony Workman, Ebubechukwu Ezeh, Samson Teka

**Affiliations:** School of Medicine Internal Residency Program, Marshall University, Huntington, WV 25701, USA; School of Medicine Internal Residency Program, Marshall University, Huntington, WV 25701, USA; School of Medicine Internal Residency Program, Marshall University, Huntington, WV 25701, USA; School of Medicine Internal Residency Program, Marshall University, Huntington, WV 25701, USA

**Keywords:** atezolizumab, hypophysitis, hypopituitarism, PD-L1 inhibitor, immune checkpoint inhibitor

## Abstract

Immune checkpoint inhibitors (ICIs) are one of the novel treatment strategies for malignancies, and their wide use has led to the emergence of immune-related adverse events (irAEs). Most of them have been reported in patients taking cytotoxic T lymphocyte-associated protein 4 inhibitors and are rarely reported among those taking programmed cell death-ligand protein 1 inhibitors. Here is a 74-year-old man who underwent treatment with atezolizumab for 33 weeks for hepatocellular carcinoma before presenting with chronic symptoms and laboratory results consistent with central adrenal insufficiency. Brain imaging did not show a possible culprit. He was incidentally found to have low thyrotropin (TSH) and low thyroxine prior to his presentation and began replacement with no further workup prior. We advocate keeping a low threshold for the diagnosis of adrenal insufficiency among patients taking ICIs and monitoring their pituitary hormones on a regular basis. Also, it is crucial to rule out pituitary hormonal deficiency among patients with central hypothyroidism prior to initiating replacement.

## Introduction

Immune cells are tailored to respond to foreign invasions, but tumor cells have found ways to escape T-cell killing mechanisms by upregulating proteins that inhibit them from triggering apoptosis. Immune checkpoint inhibitors (ICI), a class of immunotherapy, nowadays are being used for the treatment of various cancers such as melanoma, lung cancer, and prostate cancer. They are medications that block different checkpoint proteins, including cytotoxic T lymphocyte–associated protein 4 (CTLA-4), such as ipilimumab; programmed cell death protein 1 (PD-1), such as pembrolizumab and nivolumab; and programmed cell death ligand protein 1 (PD-L1), such as atezolizumab. ICIs have caused a revolution in cancer therapy and are an area of focus; nevertheless, the price for such advancements can be high: IC blockade is accompanied by a variety of side effects termed *immune-related adverse events* (irAEs). They typically resemble a variety of nonimmune diseases, such as enterocolitis and hepatitis [1]. irAEs also involve immune diseases, such as thyroid dysfunction, primary adrenal insufficiency, and type 1 diabetes, with the main concern in this article being the development of hypophysitis [1]. Cases of such associated with PD-L1 inhibitors are scarce; the rate of patients who develop hypophysitis among them can be as low as 0.5% [2].

## Case Presentation

A 74-year-old man presented to the emergency department with vague symptoms of generalized fatigue, weakness, poor oral intake, and episodes of hypotension for 3 months prior to admission. His past medical history was significant for stage 4 hepatocellular carcinoma (HCC) with adrenal metastasis status posthepatic mass cryoablation and left adrenalectomy 4 years prior. His hospital course was uncomplicated after the procedure and did not require hormonal replacement, but due to the recurrence of HCC, dual immunotherapy was initiated in October 2021 with bevacizumab (vascular endothelial growth factor inhibitor) and atezolizumab (PD-L1 inhibitor). The patient completed 33 weeks of therapy or 8 cycles, and his symptoms began roughly 21 weeks after its initiation.

## Diagnostic Assessment

In the emergency department, the patient was lethargic, confused, excessively weak, and hypotensive with maximum systolic blood pressure (BP) being 72 mm Hg. His BP temporarily responded to intravenous (IV) fluid boluses. Laboratory examination included a complete blood count, a basic metabolic panel, and a liver function test [Table 1]; the results were unremarkable except for mild hypercalcemia and hypomagnesemia, while the sodium and potassium levels were within normal range. Of note, he had multiple episodes of hypoglycemia with the lowest being 65 mg/dL (3.61 mmol/L) (reference range, 70-100 mg/dL) despite tolerating an oral diet and not being treated with glucose-lowering agents.

**Table 1.** Miscellaneous laboratory results

Laboratory testing	Results	Reference range
Hemoglobin	**13.5 g/dL (135 g/L)**	13.8-17.2 g/dL (138-172 g/L)
MCV	94.4 Fl	82-99 Fl
WBC	9.8 K/mm^3^	4.0-11.0 K/mm^3^
Platelets	148 K/mm^3^	130-400 K/mm^3^
Neutrophils	6.7 K/mm^3^	1.5-8.0 K/mm^3^
Lymphocytes	1.8 K/mm^3^	1.0-4.0 K/mm^3^
Monocytes	**1.0 K/mm^3^**	0.0-0.5 K/mm^3^
Eosinophils	**0.3 K/mm^3^**	0.0-0.2 K/mm^3^
Basophils	0.0 K/mm^3^	0.0-0.4 K/mm^3^
Na^+^	139 mEq/L (139 mmol/L)	136-145 mEq/L (136-145 mmol/L)
K^+^	4.1 mEq/L (4.1 mmol/L)	3.5-5.1 mEq/L (3.5-5.1 mmol/L)
Cl^−^	103 mEq/L (102 mmol/L)	98-107 mEq/L (98-107 mmol/L)
Ca^+2^	**10.5 mEq/L (10.5 mmol/L)**	8.4-10.2 mEq/L (8.4-10.2 mmol/L)
Ionized calcium	**5.73 mg/dL (1.43 mmol/L)**	4.6-5.18 mg/dL (1.2-1.32 mmol/L)
PO^−4^	3.5 mEq/L (3.5 mmol/L)	2.8-4.5 mEq/L (2.8-4.5 mmol/L)
Mg^+2^	**1.2 mEq/L (1.2 mmol/L)**	1.6-2.6 mEq/L (1.6-2.6 mmol/L)
CO_2_	29 mEq/L (29 mmol/L)	22-29 mEq/L (22-29 mmol/L)
BUN	23 mEq/L (23 mmol/L)	7-25 mEq/L (7-25 mmol/L)
Creatinine	1.08 mg/dL (95.47 umol/L)	0.72-1.25 mg/dL (61.9-115 umol/L)
GFR	>60 mL/min	>60 mL/min
Glucose	**65 mg/dL (3.61 mmol/L)**	70-105 mg/dL (3.9-5.5 mmol/L)
Total proteins	6.4 g/dL (64 g/L)	6.0-8.3 g/dL (60-83 g/L)
Albumin	**3.0 g/dL (30 g/L)**	3.4-5.4 g/dL (34-54 g/L)
Total bilirubin	0.6 mg/dL (10.2 umol/L)	0.1-1.2 mg/dL (5.1-17 umol/L)
Direct bilirubin	0.3 mg/dL (5.1 umol/L)	0.0-0.3 mg/dL (3.4-12 umol/L)
ALP	42 IU/L (0.714 ukat/L)	40-129 IU/L (0.68-2.45 ukat/L)
AST	20 IU/L (0.34 ukat/L)	0-35 IU/L (0.0-0.58 ukat/L)
ALT	10 IU/L (0.17 ukat/L)	10-40 IU/L (0.17-0.68 ukat/L)

Abnormal values are shown in bold font. Values in parenthesis are International System of Units (SI). Abbreviations: MCV, mean corpuscular volume; WBC, white blood count; GFR, glomerular filtration rate; ALP, alkaline phosphatase; AST, aspartate aminotransferase; ALT, alanine transaminase; BUN, blood urea nitrogen.

**Figure 1.**
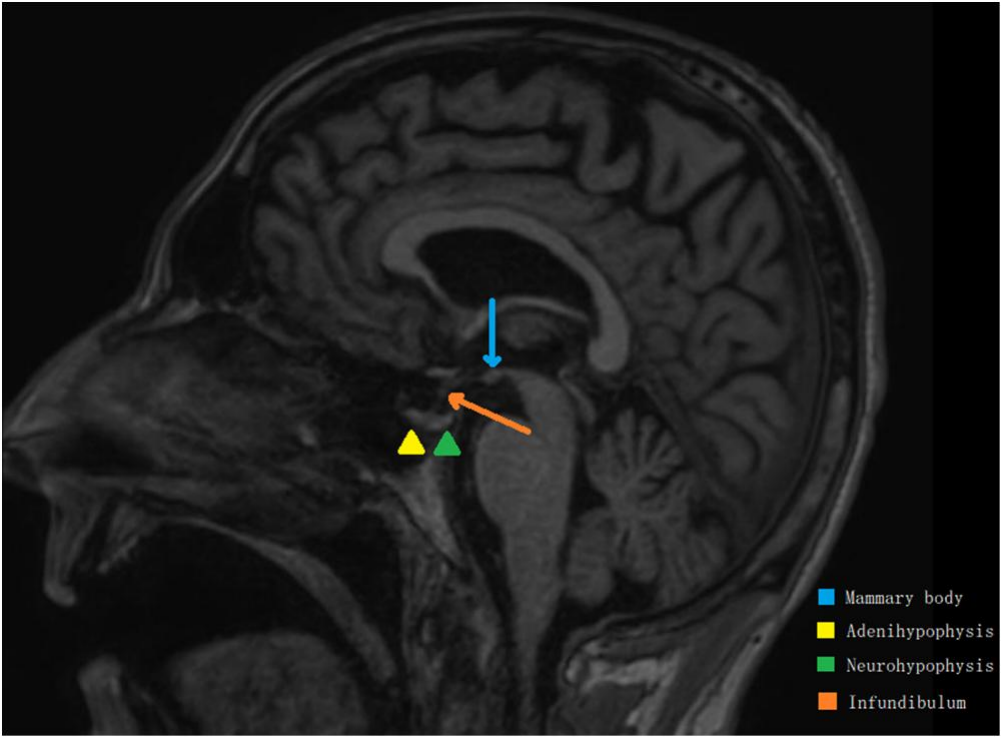
A sagittal magnetic resonance imaging of the pituitary gland showing a normal anatomy with no overt swelling, injury, or mass.

Due to our high index of suspicion, we tested for endocrine labs [Table 2]. 8 Am Cortisol level was significantly low along with the adrenocorticotropin (ACTH), and it was noticed the patient had low testosterone, normal aldosterone, and normal renin levels. No anatomical abnormalities, masses, hypoplasia, infarction, or even pituitary enhancement were found on the brain magnetic resonance imaging (MRI) shown in [Fig. 1]. On further review, we discovered that he was previously diagnosed with secondary hypothyroidism in an outside facility, evidenced by low thyroxine and TSH levels, following the fourth cycle of atezolizumab at around 14 weeks from its initiation. Oral levothyroxine was then initiated, but no further testing was conducted at that time.

**Table 2.** Endocrine laboratory testing

Laboratory testing	Results	Reference range
ACTH	**1.9 pg/mL (1.69 pmol/L)**	7.2-63.3 pg/mL (2-11 pmol/L)
Cortisol	**1.3 mcg/dL (35.86 nmol/L)**	6.2-19.4 mcg/dL (138-635 nmol/L)
Aldosterone	3.6 ng/dL (46.33 pmol/L)	0.0-30.0 ng/dL (190-830 pmol/L)
Total testosterone	**42 ng/dL (1.46 nmol/L)**	264-916 ng/dL (10-35 nmol/L)
Free testosterone	**0.55 ng/dL (0.02 nmol/L)**	5.0-21.0 ng/dL (0.17-0.73 nmol/L)
% free testosterone	**1.31% (2.5%)**	1.5-4.2%
FSH	2.5 mIU/mL	1.5-12.4 mIU/mL
LH	2.1 mIU/mL	1.7-8.6 mIU/mL
IGF-1	73 ng/mL	64-240 ng/mL
Prolactin	11.1 ng/mL (11.1 ug/L)	4.0-15.2 ng/mL (4.0-15.2 ug/L)
Thyroxine free	1.21 ng/dL (15.57 pmol/L)	0.70-1.48 ng/dL (9.0-19.0 pmol/L)
Plasma renin activity	2.865 ng/dL (0.68 pmol/L)	0.167-5.380 ng/dL (0.04-1.27 pmol/L)

Abnormal values are shown in bold font. Values in parenthesis are International System of Units (SI). Abbreviations: ACTH, adrenocorticotropic hormone; FSH, follicle-stimulating hormone; LH, luteinizing hormone; IGF, insulin-like growth factor.

## Treatment

The patient was started on hydrocortisone 100 mg IV loading dose and 50 mg IV doses every 6 hours for 48 hours total.

## Outcome and Follow-up

Since starting the medication, his BP and glucose readings remained within an acceptable range, and the symptoms dramatically improved. The patient showed a steady return of his strength as we switched him to an oral, maintenance dose of hydrocortisone 10 mg in the evening and 20 mg in the morning with complete resolution of his confusion and adequate correction of the electrolyte abnormalities.

## Discussion

The mechanism by which hypophysitis occurs due to ICI treatment is not well understood yet, but one hypothesis suggests that it occurs secondary to T cell–mediated immune destruction [1]. Another article supports the idea that it starts with the development of antibodies targeting ACTH, as it revealed that more than half of the patients presenting with acquired isolated ACTH deficiency carry anticorticotropin antibodies [3]. This is different from the pathogenesis of autoimmune hypophysitis (AH), which is an inflammatory disorder of the pituitary gland caused by infiltration of the gland by immune cells and the corresponding mass effect [4]. The incidence can reach up to 17% among patients taking CTLA-4 inhibitors [5], with the range of onset of symptoms being 6 to 11 weeks after starting the medication [6]. With patients taking PD-L1 inhibitors the frequency of hypophysitis is much less, occurring approximately 10 to 46 weeks after starting the medication [6].

Our patient had a history of HCC prompting a long course of atezolizumab for 33 weeks, which raises the suspicion for irAEs. His history of metastasis to the adrenal gland and surgical removal could be a contributing factor, but his postoperative course was unremarkable, and his hormonal levels were within normal range prior to starting the immunotherapy. Patients with adrenal insufficiency frequently present with nonspecific symptoms; our patient presented with fatigue, anorexia, and recurrent episodes of hypotension, and other reported symptoms can include nausea, headaches, vision disorders, and it can even be asymptomatic [6].

Laboratory test results were typical of that of central adrenal insufficiency; we saw a deficiency of ACTH along with hypocortisolism. Also, it was incidentally discovered that the patient was deficient both in thyroxine and TSH indicating a central cause, but there were no further investigations afterward. It is of great importance to work up central causes of hypothyroidism; in fact, it is strongly recommended in guidelines to evaluate patients with central hypothyroidism for ACTH deficiency before starting thyroxine therapy, which may initiate an adrenal crisis [7]. Pituitary hormonal deficiency is based on the anatomical area the infiltration has involved; however, ACTH and TSH deficiencies are frequent in early stages of ICI hypophysitis, unlike other causes of hypopituitarism [8]. It is important to know that the ACTH stimulates the 2 innermost layers of the adrenal cortex, the zona fasciculata and zona reticularis, sparing the outermost, the zona glomerulosa. Thus, ACTH deficiency will result in a shortage in the production of glucocorticoids, produced from the zona fasciculata, and androgens, produced from zona reticularis, ultimately leading to a drop in BP, glucose levels, and testosterone levels, as was manifested in our case. The idea that the zona glomerulosa is not stimulated by the ACTH explains why the aldosterone and renin levels are preserved, maintaining a normal balance in the plasma sodium and potassium levels. Mild hypercalcemia was noted in the laboratory results, which could be a manifestation of volume depletion, but it could also be linked to adrenal insufficiency in mechanisms that are not fully understood in detail.

Comparing AH and hypophysitis secondary to ICI, presentation is often similar; however, visual problems and headaches are less likely to be present in ICI hypophysitis. Imaging modalities like MRI can be used to exclude other etiologies such as neoplasm or AH. It was thought MRI is often not contributive to diagnosing ICI hypophysitis, as no changes are readily seen; however, prior cases of ICI-induced hypophysitis have shown different findings: Almost half of these patients can have diffuse enlargement of the pituitary and the stalk, but other findings can include hypoenhancing lesions on the anterior lobe and even empty sella syndrome may develop in the long term. It has been noticed that these MRI changes are seen more among patients taking CTLA-4 inhibitors than PD-L1 inhibitors. While the regular use of MRI to diagnose hypophysitis among patients taking ICIs may not be recommended, the use of this modality can help identify other possible causes when symptoms arise. Also, many cases of ICI-induced hypophysitis have had normal MRI findings of the pituitary gland, thus, a normal MRI does not exclude the diagnosis of hypophysitis [8, 9].

Given the patient's older age, male sex, and lack of prior autoimmune disease, it was highly unlikely that this would be a case of AH. In addition, there was a lack of clear clinical evidence of an alternative clinical culprit, both on imaging and laboratory testing, and there was a clear temporal relationship and presentation like prior cases of PD-L1–induced hypophysitis, leading to a determination that the patient was indeed suffering from atezolizumab-induced hypophysitis. Due to the severity of potential outcomes of pituitary dysfunction, the fact that all the cases of ICI-induced hypophysitis were associated with adrenal insufficiency [10], and the increasing reports make it reasonable to earnestly consider testing before symptom onset.

In conclusion, given the growth of the field of immunotherapy, clinicians should be aware of irAEs and learn further about these medications and their potential toxicities. It is utterly important to identify cases of adrenal insufficiency in such patients given the severity of its outcomes, which can potentially lead to hypotension and hypoperfusion, as well as the impairment of the quality of patients’ lives and intolerance to cancer treatment. We believe clinicians should keep a watchful eye on the development of such adverse effects. Regular follow-up of electrolytes and hormones, especially 8 Am cortisol level and pituitary hormones, should be prompted for every patient initiating ICIs. Endocrine adverse effects can be adequately managed by simply replacing the deficiency, which does not contraindicate the continued use of immunotherapy.

## Learning Points

- Keep a low level of threshold for the diagnosis of adrenal insufficiency in patients treated with ICIs, given the severity of such conditions. Usually, they present with nonspecific symptoms and require a high level of suspicion to be able to diagnose them.
- Central hypothyroidism, in which both the TSH and thyroxine are low, should prompt further workup to rule out hypopituitarism and adrenal insufficiency prior to starting levothyroxine replacement.
- Regularly monitor electrolytes and pituitary hormones for all patients starting on ICIs. Guidelines recommend obtaining baseline levels and then monitoring monthly for the first 3 to 6 months, then it can be spaced out every 3 months, 6 months, and annually.

## Data Availability

Data sharing is not applicable to this article as no data sets were generated or analyzed during the current study.

## Abbreviations

ACTH: adrenocorticotropin
AH: autoimmune hypophysitis
BP: blood pressure
CTLA-4: cytotoxic T lymphocyte–associated protein 4
HCC: hepatocellular carcinoma
ICI: immune checkpoint inhibitor
irAE: immune-related adverse event
IV: intravenous
MRI: magnetic resonance imaging
PD-L1: programmed cell death ligand protein 1
TSH: thyrotropin
